# Progress in Medicine

**Published:** 1895-04-13

**Authors:** 


### Progress in Medicine.

### DISEASES OF THE CIRCULATORY SYSTEM.

*(Continued from page 10.)*

**Aneurism.**—A new diagnostic sign of aneurism of the aorta[1] is to be found in the presence of a systolic sound in the brachial artery, sometimes accompanied with an arterial murmur. When aortic regurgitation is present, however, this sound may exist without any aneurism, otherwise it is of great value as an early symptom. The fluid pressure on the total area of an aneurism, of course, varies as the area, but it has been pointed out by Dawson Turner[2] that the pressure per square inch is also greater than in the normal vessel. "Where the velocity is greatest there the pressure is least. A particle of fluid entering the wider part of a tube finds a greater pressure in front of it resisting its advance than behind it urging it on." There is thus not only very great pressure on the walls of an aneurism, which causes its gradual distension, but the work of the heart is increased, since the aneurism dams back the blood. We cannot, therefore, look on an aneurism as in any sense a safety valve.

Some large intra-cranial aneurisms in young people were shown at the Clinical Society. It was pointed out that usually they are not larger than a pea,[3] and that subretinal hæmorrhages were almost unknown, whereas in one of these cases there were in both eyes large well marked ones. Fatal bleeding from an aneurism of the hepatic artery was caused by gallstones in a woman of forty years of age. Four hæmorrhages took place in five weeks.[4] Three gallstones were found, the pressure of which had caused an opening into the right branch of the hepatic artery. Douglas Powell, in discussing the symptoms of abdominal aneurisms,[5] remarks that a bruit is one of the least important and most misleading of all diagnostic points. It may occur without an aneurism, and in half the cases of this affection at the time when a diagnosis has to be made, there is no bruit at all. Thrill and bruit again may occur without aneurism, and if communicated through a solid tumour the symptom may disappear when the patient is placed in the prone or knee-elbow position, so that the aorta is relieved of the weight of the tumour. "Collateral evidence, too, of the unsoundness of vessels is of great weight in the diagnosis of a tumour as aneurismal." Thus an aortic diastolic murmur is of great importance in showing a condition of the arteries likely to produce an aneurism. In discussing a case of thoracic aneurism he laid stress on the altered voice, the brassy laryngeal cough, the presence of a small area of dulness over which was an impulse perceptible by the ear alone, with a peculiar diastolic jog, pains in the shoulder, attacks of dyspnœa, and tracheal tugging felt in the neck.

**Sudden Death in Heart Disease.**—In connection with the occurrence of sudden death from aneurism, it may be useful to consider the various ways in which sudden death is produced by disease of the heart. Councilman enumerates these as follows: (*a*) Endocardial thrombosis and embolism; (*b*) lesions of the walls chiefly from disease of the coronary arteries, such as infarction, intestinal myocarditis, and aneurism, necrotic and fatty degeneration; (*c*) aortic stenosis, and rarely pericarditis.[6]

**Indurative Mediastino Pericarditis.**—Much difficulty is found in the diagnosis of indurative mediastino pericarditis, but an exhaustive discussion of the subject is given by T. Harris with references to the literature.[7] Pathologically we find an adherent pericardium, with an increase of fibroid tissue in the mediastinum, which is united to the outer side of the pericardium, while the latter and the fibroid tissue are both united to the lungs. There may be also caseous degeneration of the lymphatic glands. Strictly, we should separate from it cases where there are external and internal pericardial adhesions without increase of the fibroid tissue, and cases of this increase without pericardial adhesions. Since Kussmaul, in 1873, drew attention to the occurrence of the pulsus paradoxus and the distension of the cervical veins during inspiration in this affection, these two signs together have been regarded as diagnostic of indurative med. pericarditis. Especially has stress been laid on the distension of the right external jugular vein when a deep inspiration is taken, but Harris shows that this swelling of the veins is undoubtedly absent in some cases. Besides these symptoms, then, we may find cardiac enlargement, venous engorgement and cyanosis, dyspnœa, enlargement of liver, ascites, and sometimes general dropsy. The heart sounds may be natural, or affected from other causes. Ascites is sometimes one of the chief features of the disease, and may be due to the chronic inflammation of the liver or to a special chronic or to a tubercular peritonitis. The ascites with little or no general œdema appears to be often due to this special form of chronic peritonitis which again has no connection with that of granular kidney. The pulsus paradoxus, where there is lessening or disappearance of the pulse of all arteries during the inspiratory act while the action of the heart remains regular, is certainly not confined to cases of this affection, but is frequently met with in it and is produced by the dragging of the adhesions on the aorta and in other ways. There are, indeed, no signs absolutely diagnostic of the disease when taken by themselves, but an approach to certainty may be made when several of them occur together. Its duration may be several years and, unfortunately, nothing can be done in the way of treatment beyond general care of the heart whose action is thus interfered with.

**Paracentesis of the Pericardium.**—A remarkable case in which paracentesis pericardii was performed twice with relief, twenty-eight ounces of fluid being removed on the second occasion, was reported by Percy Kidd,[8] who thinks the operation may safely be undertaken, not only in acute affections but whenever in the later stages of dropsy the pericardial fluid is adding to the difficulties of the heart. Marmaduke Shield dwelt on the difficulty of distinguishing an effusion from an enlarged heart with adherent pericardium. In view of the danger of injuring the wall of the heart in operating, he insisted that an incision should first be made in the skin and the trocar then passed obliquely. Ewart agreed with Dr. Kidd on the advisability of the operation, but preferred laying the pericardium open to aspirating it. In a case of rheumatic pericarditis the pulse and respiration had ceased, and a hurried attempt by A. T. Sloan at paracentesis being made, the heart itself was tapped and blood withdrawn.[9] This sufficed to recall it to activity, though in a rapid and tumultuous fashion. It finally steadied down, and a complete recovery followed. The author of the paper thinks that in some conditions of heart failure it would be useful to tap the right ventricle by a hypodermic syringe.

**Pain in Cardiac Failure.**—Pain and hyperæsthesia of certain sensory nerves may afford valuable indications of heart failure where few or no physical signs are present. The delimitation of the spinal nerve areas associated with the visceral nerves supplying the heart has been carefully worked out by J. Mackenzie.[10] They are the spinal accessory, the second and third cervical, and the group from the seventh cervical to the fifth dorsal. The area of hyperæsthesia can be determined by drawing the head of a pin along the skin, or in a rapid examination by pressing over the second rib in the nipple line, over the trapezius above the spine of the scapula, again over the upper dorsal vertebræ, and by pinching the left sternomastoid muscle. If sensory phenomena are present, they can generally be detected in one or all of these spots. Thus, where bronchitis is mainly dependent on heart failure, this cardiac area of hyperæsthesia will be marked, and affords a valuable guide to treatment. The writer throws out the suggestion that herpes zoster may be produced by visceral irritation acting on allied spinal nerves in the same way as imitations of the vagus and sympathetic from the heart cause these areas of hyperæsthesia through the nerves mentioned above.

**Functional Heart Disease.**—In the discussion on functional diseases of the heart at the Bristol meeting[11] Douglas Powell classified them as follows: (*a*) Cardio-vascular hyperæsthesias—undue perception of the heart's action, oppressed action, and anginas, produced by vaso-motor irritation or variable degrees of arterial tension. (*b*) Accelerated action from mental excitement, vagus irritation, vaso-motor relaxation, direct irritation, &c. (*c*) Retarded action, sustained or paroxysmal, occasionally congenital, but largely due to vaso-motor and vagus irritation through the poisons of previous exanthemata. His view generally appears to be that most of these affections appear to depend on changes, spasms or relaxations of the vaso motor mechanism altering seriously the amount of work to be met by the heart. A large proportion of the cases of angina depend on a functional vaso-motor spasm of this kind increasing the intra-cardiac pressure. Indeed, the development of mitral incompetence sometimes puts a stop to anginous attacks. Paroxysmal tachycardia is another type of purely nervous affection, combated most successfully by nerve remedies, and in which heart changes are merely secondary. Closely allied to it is Graves' disease, which is essentially a functional hurry of the heart due to vaso-motor paralysis. P. Chapman pointed out that functional alterations may in some cases depend on organic changes. The short contractions of fatty degeneration are an instance. Then again irritation of the vagus increases the power and work of the heart, for the vagus is a trophic nerve and nourishes and builds up the tissue of the heart. Section of the vagus causes fatty degeneration of the heart in birds and rabbits. The accelerator nerves are katabolic or destructive of tissue. Thus in the tachycardia of Graves' disease the heart dilates, wastes, and becomes weaker. On the whole then we must not regard functional diseases as coinciding with those of nervous origin. We may have disease of nerve or muscle without valvular or other strictly structural disease.

**Lymph Formation.**—The question of the formation of lymph has undergone much discussion. Heidenhain[12] appeared to have proved that certain substances, such as crab's muscle, cause a direct overflow of lymph from the vessels without any effect from pressure, that in fact lymph is due to a secretion of the fixed cells of the capillary wall and not to any filtration under pressure. His results have been overthrown by his pupil, E. H. Starling, who by elaborate experiments shows that really an increase of pressure and permeability are the true source of the action, even of these so-called lymphagogues, and that the nervous system too has no direct influence, but only through increasing intra-capillary pressure. However, he confesses that before we can definitely accept the pressure theory of transudation we must explain how it is that after the injection of sugar, salt, and peptones, the percentage of these bodies in the the lymph is higher than in the blood plasma.

[1] B.M.J., Nov. 17. [2] Ed. Med. J., Oct. [3] Lanc., Oct. 20. [4] Am. J. Med. Sc., Nov. [5] Clin. J., Aug. 22. [6] Am. J. Med. Sc., Nov. [7] Med. Chronic., Oct.- Feb. [8] Lanc., Feb. 2, '95. [9] Ed. Med. J., Feb., '95. [10] Lanc., July 5. [11] B.M.J., Nov. 11. [12] Med. Chron. Nov.

